# Magnetic CoFe_2_O_4_ and NiFe_2_O_4_ Induced Self-Assembled Graphene Nanoribbon Framework with Excellent Properties for Li-Ion Battery

**DOI:** 10.3390/molecules28104069

**Published:** 2023-05-12

**Authors:** Xiyu Zhao, Chunyang He, Qiujv Bai, Xiangwen Miao, Cheng Cao, Tianli Wu

**Affiliations:** 1 Key Laboratory of Hexi Corridor Resources Utilization of Gansu, School of Chemistry and Chemical Engineering, Hexi University, Zhangye 734000, China; 18716252366@163.com (X.Z.); 18993575652@163.com (C.H.); w32579778581106@163.com (Q.B.); 18153875414@163.com (X.M.); caochxu@126.com (C.C.); 2 Henan Key Laboratory of Photovoltaic Materials, Henan University, Kaifeng 475001, China

**Keywords:** CoFe_2_O_4_, NiFe_2_O_4_, graphene nanoribbons, Li battery, magnetism

## Abstract

A magnetically induced self-assembled graphene nanoribbons (GNRs) method is reported to synthesize MFe_2_O_4_/GNRs (M = Co,Ni). It is found that MFe_2_O_4_ compounds not only locate on the surface of GNRs but anchor on the interlayers of GNRs in the diameter of less than 5 nm as well. The in situ growth of MFe_2_O_4_ and magnetic aggregation at the joints of GNRs act as crosslinking agents to solder GNRs to build a nest structure. Additionally, combining GNRs with MFe_2_O_4_ helps to improve the magnetism of the MFe_2_O_4_. As an anode material for Li^+^ ion batteries, MFe_2_O_4_/GNRs can provide high reversible capacity and cyclic stability (1432 mAh g^−1^ for CoFe_2_O_4_/GNRs and 1058 mAh g^−1^ for NiFe_2_O_4_ at 0.1 A g^−1^ over 80 cycles).

## 1. Introduction

Rechargeable lithium-ion batteries (LIBs) are widely used to meet the ever-increasing demand for fast charging and high-capacity energy storage [1,2,3]. It is particularly important to search for novel anode materials with both high capacity and outstanding safety for high-performance LIBs. Nowadays, graphite is the most commercial anode material for LIBs owing to its abundant natural resources. However, the relatively low theoretical specific capacity (372 mAh g^−1^) and the lithium diffusion coefficient (10^−7^~10^−10^ cm^2^ s^−1^) restrict the further application of high-power LIBs [4,5,6]. The choice of magnetic transition metal oxides (TMOs) as anodes for LIBs has received considerable attention due to their high energy density and natural abundance. Apart from binary TMOs, ternary TMOs comprising mixed valence in a single crystalline structure have drawn enormous research attention [7,8]. Regarding cobalt ferrite and nickel ferrite (MFe_2_O_4_, M = Co, Ni), as one of the representative ternary TMOs, better electrical conductivity arises because Co^2+^/Ni^2+^ and 1/2 the Fe^3+^ cation occupy the octahedral sites. In comparison to conventional spinel oxides, MFe_2_O_4_ with a unique inverse spinel structure has higher Li^+^ ion incorporation [9,10]. As a result, MFe_2_O_4_ is expected to become an appealing anode material for LIBs. Nevertheless, MFe_2_O_4_ suffers from huge volume expansion and material cracking problems during the periodic charge–discharge process, which results in rapid fading capability [11,12]. Two common approaches have been proposed to address the above issues. One is to prepare variously nanostructured (such as hollow nanospheres, nanowires, nanotubes) [13,14,15,16] composite. Nanostructured MFe_2_O_4_ can shorten the diffusion paths of Li^+^ ion, leading to higher electrochemical activity.

The other route is to introduce carbon materials, such as carbon nanotubes (CNTs) and graphene, to fabricate MFe_2_O_4_/carbon composites [17,18,19]. Among them, nest structure MFe_2_O_4_/carbon composites, due to their porous structure, can increase the efficiency of electron transport and the contact area with electrolytes. Moreover, a network carbon framework can not only effectively accommodate volume change in the MFe_2_O_4_ and prevent the pulverization, which will enable nest MFe_2_O_4_/carbon composites to maintain high conductivity and reversible capacity, but also provide nucleation sites that regulate MFe_2_O_4_ crystal morphology [20,21,22,23].

Nest structure MFe_2_O_4_/carbon composites can be prepared by hydrothermal, chemical vapor deposition, polymer-assisted methods, and so on [24,25,26]. The 3D framework prepared by the polymer-assisted method is stable in structure and simple in operation. However, the poor-conductivity nature of the polymer would affect the electron transport among the composites. Nest MFe_2_O_4_/carbon composites with high electron conductivity can be obtained by replacing the polymer with magnetic MFe_2_O_4_; thus, MFe_2_O_4_ will not only act as the active material but also as the “binder agent” to stabilize the carbon framework. The common carbon framework is mainly created by graphene or CNTs [26,27]. Compared to them, graphene nanoribbons (GNRs) are more beneficial for improving the electrochemical performance of the composites owing to their high active open edges and specific surface area [28].

Based on our previous work [29], a facile magnetically induced self-assembled graphene nanoribbons (GNRs) method is reported to synthesize MFe_2_O_4_. The key feature for our synthesis route is taking advantage of the magnetic attraction of MFe_2_O_4_, which cross-link GNRs in the intersections. By means of this procedure, the self-aggregated phenomenon takes place in magnetic nanomaterials owing to more active sites at the GNRs’ junction; MFe_2_O_4_ particles tend to link to each other and accumulate at the joints. After a following heating treatment, MFe_2_O_4_/GNRs can be easily achieved. MFe_2_O_4_/GNRs present excellent cycle performance or LIBs and particularly excellent high reversible capacity and rate capacity, resulting from their unique network structure and the synergy effect of each component. Additionally, research on magnetism indicates that the MFe_2_O_4_/GNRs are also promising for magnetic devices.

## 2. Results and Discussion

### 2.1. SEM and TEM Analysis

**Figure 1:**
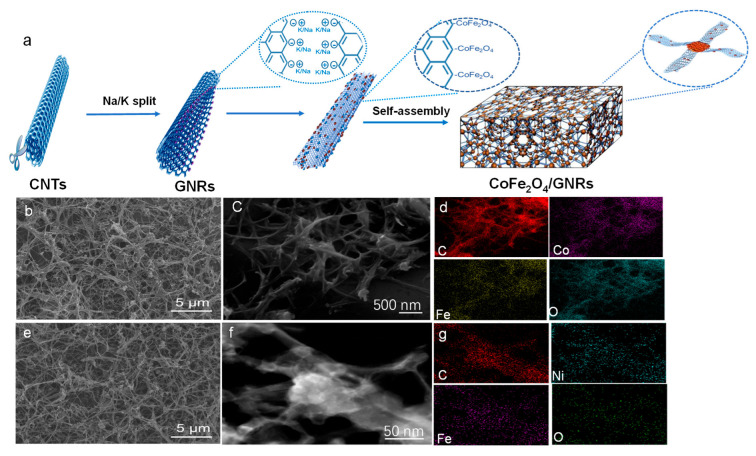
(**a**) The schematic illustration of the synthesis of CoFe_2_O_4_/GNRs composites. (**b**,**c**) SEM images of as-prepared CoFe_2_O_4_/GNRs and (**d**) EDS mapping of CoFe_2_O_4_/GNRs. (**e**,**f**) SEM images of NiFe_2_O_4_/GNRs and (**g**) EDS mapping of NiFe_2_O_4_/GNRs.

Figure 1a illustrates the synthesis route of CoFe_2_O_4_/GNRs composites by simple refluxing. CNTs were unzipped to obtain GNRs [30]. After that, both GNRs and cobalt organic salt were dispersed in benzyl ether solution under agitation. Thereafter, aqueous solution was refluxed at 220 °C for 0.5 h, during which Co(acac)_3_ decomposed to some CoFeO nanoclusters. Then, borders of GNRs filled with dangling C-C bonds were occupied by the CoFeO nanoclusters. Along with the reaction, CoFeO nanoclusters in situ grew and agglomerated as CoFe_2_O_4_ in the surface, interlayers, and edges of GNRs. After drying, the magnetically induced attraction of CoFe_2_O_4_ began to focus on the GNRs joints, causing CoFe_2_O_4_/GNRs composites to self-assemble. The synthesis procedure of NiFe_2_O_4_/GNRs is similar to CoFe_2_O_4_/GNRs, except nickel organic is used. The structure of the products was first investigated by SEM. Figure 1b–g represents the SEM images for MFe_2_O_4_/GNRs composites. The hybrids possessed a porous structure in the low-magnified SEM. From the high-magnification SEM (Figure 1c,f), the surface of the GNRs was anchored by metal oxide nanoparticles, and it can be clearly seen that some aggregates of MFe_2_O_4_ were in the junctions of GNRs. The aggregates of MFe_2_O_4_ acted as a “crosslink agent” that fitted together the GNRs to build a 3D porous structure. As presented in Figure 1b,e, the morphology of CoFe_2_O_4_/GNRs and MFe_2_O_4_/GNRs seemed very similar, indicating that preparation of porous MFe_2_O_4_/GNRs was controllable and universal. The SEM images of CoFe_2_O_4_ and NiFe_2_O_4_ nanocomposite without GNRs are shown in Figure S1 (Supplementary Materials). CoFe_2_O_4_ and NiFe_2_O_4_ are irregular spheres (20–50 nm) and tend to agglomerate together to form large particles without GNRs. The elemental mapping images of MFe_2_O_4_/GNRs are shown in Figure 1d,g, demonstrating that the C, Ni, Co, Fe, and O are gathered in the junction of GNRs and distributed uniformly along GNRs.

**Figure 2:**
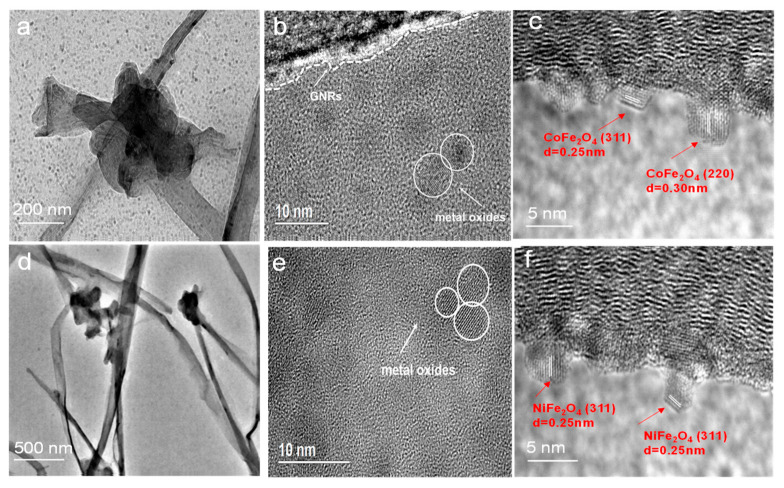
TEM images of as-prepared (**a**–**c**) CoFe_2_O_4_/GNRs and (**d**–**f**) NiFe_2_O_4_/GNRs.

The microstructure and composition of the MFe_2_O_4_/GNRs were confirmed by the TEM. As shown in Figure 2a,d, there are numerous agglomerated metal oxide nanoparticles at the joints of GNRs. With the cross-linking function of these metal oxide nanoparticles, the GNRs self-assembled into a 3D structure. Additionally, it can be observed from Figure 2b,e that the MFe_2_O_4_ in an average size of 3–8 nm was uniformly distributed on both exteriors and interior layers of the GNRs in the hybrids (Figure S2). It was noted that suspended particles of MFe_2_O_4_ were found at the borders of GNRs in Figure 2c,f. It was proven that the unsaturated C-C bond at the edge of the GNRs can facilitate the nucleation and growth of MFe_2_O_4_. Figure 2c showed the high-resolution lattice images of CoFe_2_O_4_/GNRs nanoparticles in CoFe_2_O_4_/GNRs composite. The lattice fringes with a lattice spacing of 0.30 nm and 0.25 nm corresponded to the (220) and (311) planes of CoFe_2_O_4_, respectively [31]. Figure 2f showed the high-resolution lattice images of NiFe_2_O_4_ nanoparticles. The lattice fringes with a lattice spacing of 0.25 nm were assigned to the (311) planes of NiFe_2_O_4_ [32].

### 2.2. XRD, Raman, and XPS Analysis

**Figure 3:**
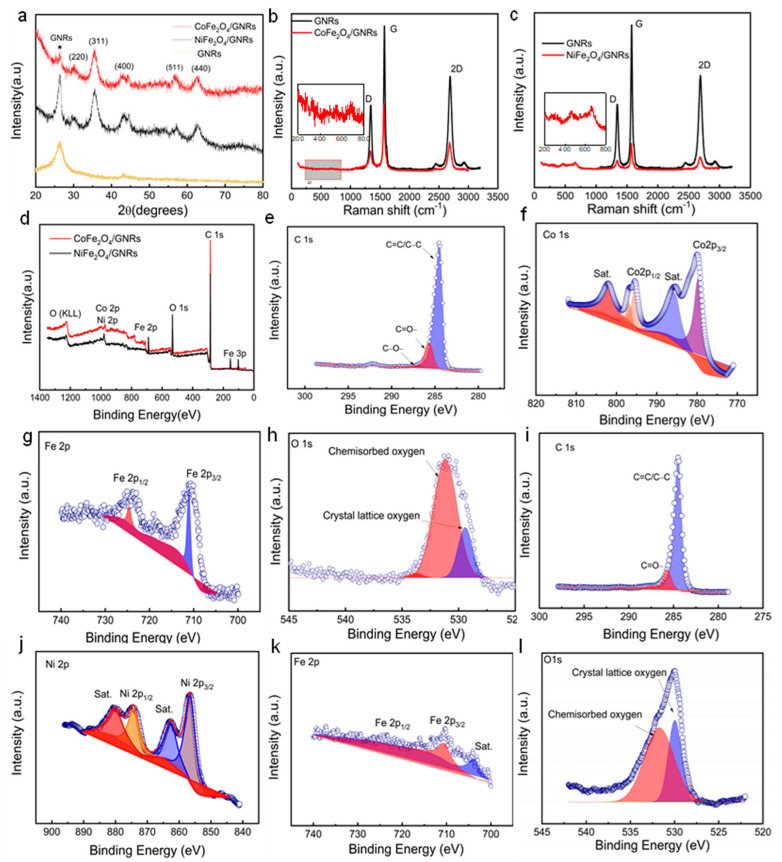
(**a**) ★ Represents the XRD peaks of graphene nanoribbons (GNRs); XRD patterns and Raman spectra of (**b**) CoFe_2_O_4_/GNRs and (**c**) NiFe_2_O_4_/GNRs. (**d**) Full XPS spectra of as-prepared CoFe_2_O_4_/GNRs and NiFe_2_O_4_/GNRs. XPS survey spectra of CoFe_2_O_4_/GNRs (**e**) C 1s. (**f**) Co 2p. (**g**) Fe 2p. (**h**) O1s and NiFe_2_O_4_/GNRs. (**i**) C 1s. (**j**) Ni 2p. (**k**) Fe 2p. (**l**) O1s.

An X-ray diffraction (XRD) analysis was performed to assess the structure, crystallization, and purity of MFe_2_O_4_/GNRs. As shown in Figure 3a, for CoFe_2_O_4_/GNRs composite, the diffraction peaks at 18.3, 30.2, 35.5, 43.2, 57.1, and 62.7 correspond to the lattice planes (220), (311), (400), (511), and (440), respectively, of cubic spinel structure (JCPDS No. 22-1086 and NiFe_2_O_4_ with JCPDS card no. 86-2267). In addition, there was also a diffraction peak at 26.5°(star symbol in Figure 3a), which was assigned to the GNRs [31,32]. Raman measurement was performed in Figure 3b,c. D, G, and 2D bands at 1340, 1582, and 2678 cm^−1^ stand for GNRs [32] on account of the strong disorder carbon’s in-plane vibrations and graphitic carbon’s in-plane vibrations. The three peaks of GNRs in MFe_2_O_4_/GNRs are basically in the same position as those in GNRs alone, but the strength of all three peaks is significantly reduced since GNRs are combined with MFe_2_O_4_. In the enlarged part of the figure, both the composites show three major peaks. CoFe_2_O_4_ shows at 680, 532, and 320 cm^−1^. Further, the peaks of NiFe_2_O_4_ are at 672, 470, and 323 cm^−1^ due to the low Raman-intensity nature of MFe_2_O_4_ [33,34], which are all consistent with the XRD results. XPS measurements were performed to further study the surface chemical state of the composites (Figure 3d). For MFe_2_O_4_/GNRs, there were several major peaks, assigned to C, O, Fe, Co, and Ni, respectively. In the high-resolution XPS spectra, C_1s_ of CoFe_2_O_4_/GNRs had a graphitic C-C/C=C peak at 286.7 eV, together with C=O (287.4 eV), as revealed in Figure 3e [32]. The C-C/C=C and C-O peak of NiFe_2_O_4_/GNRs are located at 285.4 eV and 287.6eV, respectively (Figure 3i). The Co _2p3/2_ and Co_2p1/2_ peaks of the CoFe_2_O_4_/GNRs were located at 779.4 and 795.8 eV with a 16.4 eV peak-to-peak separation (Figure 3f), indicating the Co(II) oxidation state [35]. The locations of the Fe_2p_ peaks (Fe_2p1/2_ at 724.5 eV and Fe_2p3/2_ at 710.4 eV) in Figure 3g verify the presence of Fe^3+^ cations in CoFe_2_O_4_. The O_1s_ spectrum in Figure 3h demonstrates two oxygen attributions. The peaks of 529.7 and 531.2 eV represent a metal-oxygen bond and some surface defect sites, respectively. The deconvolution of the Ni_2p_ spectrum demonstrates four peaks in Figure 3j. At binding energies of 855.7 eV and 876.3 eV are two main peaks followed by two shake-up satellites. Moreover, the signals of Fe_2p3/2_ and Fe_2p1/2_ in NiFe_2_O_4_ peaks were found at 724.7 eV and 711.9 eV, respectively, indicating that the Fe is in the typical trivalent oxidation state (Figure 3k). Peaks of O_1s_ of NiFe_2_O_4_ are denoted as crystal lattice oxygen and chemisorbed oxygen in Figure 3l [36,37].

### 2.3. BET and TGA Analysis

The nitrogen adsorption-desorption isotherm and pore-size distribution curves are shown in Figure S3a,b. The Brunauer-Emmett-Teller (BET) specific surface areas of CoFe_2_O_4_/GNRs and NiFe_2_O_4_/GNRs were measured to be 47.7 m^2^g^−1^ and 27.5 m^2^g^−1^, respectively. The sample exhibits a representative type IV curve, indicating a mesoporous structure with distributions ranging from 2 to 10 nm. The foam structure will help the rapid travel of electrolytic ions for accessing the interior MFe_2_O_4_ in the lithiation-delithiation process.

The TGA curves for MFe_2_O_4_/GNRs were shown in Figure S4. TGA is performed to analyze the mass ration of MFe_2_O_4_ and GNRs. There were several stages of weight loss in Figure S4. The drop in weight below 200 °C can be ascribed to the evaporation of water. The typical weight loss between 200 °C and 550 °C can be ascribed to the oxidation of the GNRs. The one after 550 °C was mainly due to the oxidation of MFe_2_O_4_ in air. Based on the weight loss in Figure S4, the weight percentages of CoFe_2_O_4_ and NiFe_2_O_4_ in MFe_2_O_4_/GNRs were estimated to be ~41% and ~23%, respectively.

### 2.4. Electrochemistry Performance

**Figure 4:**
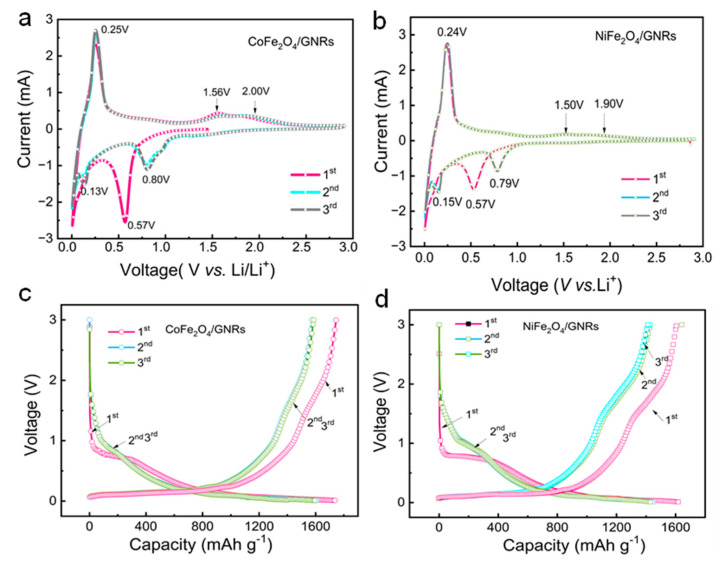
CV curves of (**a**) CoFe_2_O_4_/GNRs, (**b**) NiFe_2_O_4_/GNRs electrodes, (**c**) CoFe_2_O_4_/GNRs, and (**d**) NiFe_2_O_4_/GNRs electrodes at a current density of 0.1 A g^−1^ for the 1st, 2nd, and 3rd cycles in the voltage range between 3.00 and 0.01 V.

Cyclic voltammetry (CV) tests were investigated in half-cell configurations to assess the Li^+^ ion storage properties of the as-prepared MFe_2_O_4_/GNRs. Figure 4a–b showed the CV curves of the CoFe_2_O_4_/GNRs and NiFe_2_O_4_/GNRs in the first three cycles. Two cathodic peaks were observed at 0.57 and 0.53 V in the first cycle, corresponding to multistage electrochemical reduction reactions (lithiation) of MFe_2_O_4_/GNRs with a Li^+^ ion and a partially irreversible solid electrolyte interphase (SEI) layer [32]. The main anodic peaks at 1.5–2.0 V for the two composites were ascribed to the oxidation reactions (delithiation) of MFe_2_O_4_; in addition, the lithiation and delithiation peaks of GNRs were at 0.13–0.25 V and 0.15–0.24 V, proving that GNRs were helpful for Li^+^ ion storage. The above reactions are as follows [38,39,40,41]:

CoFe_2_O_4_ + 8Li^+^ + 8e^−^↔ 2Fe + Co + 4Li_2_O (1)

NiFe_2_O_4_ + 8Li^+^ + 8e^−^ ↔ 2Fe + Ni + 4Li_2_O (2)

6C + xLi^+^ + xe^−^↔ Li_x_C_6_ (3)

Figure 4c,d displays the charge-discharge process of MFe_2_O_4_/GNRs at a current density of 0.1 A g^−1^. In agreement with the CV curves, the potential platform at ~0.79 V can be found during the first discharge (lithiation) process. CoFe_2_O_4_/GNRs and NiFe_2_O_4_/GNRs electrodes delivered initial discharge capacities of 1735 mAh g^−1^ and 1621 mAh g^−1^and initial charge capacities of 1573 mAh g^−1^ and 1502 mAh g^−1^, with cycle efficiency of 90% and 92%, respectively. The irreversible capacity losses of the two electrodes in the following cycles were probably associated with the formation of SEI and irreversible lithium loss. It was observed that the second and third cycles of the current curve almost overlapped with each other, proposing that the Li^+^ ion storage of the electrodes was reversible in the following cycles (Figure S5).

**Figure 5:**
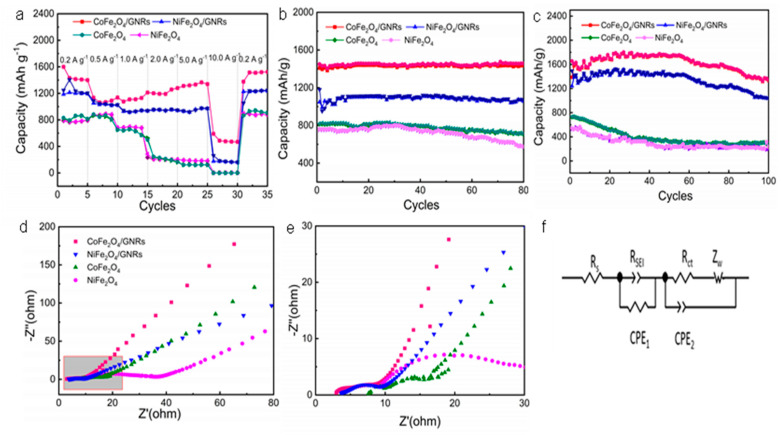
(**a**) Rate capability of the CoFe_2_O_4_, NiFe_2_O_4_, CoFe_2_O_4_/GNRs, and NiFe_2_O_4_/GNRs electrodes from 0.2 A g^−1^ to 10 A g^−1^. (**b**) Cycling performance of four electrodes at 0.1 A g^−1^. (**c**) Cycling performance of four electrodes at 1.0 A g^−1^. (**d**) Nyquist plots of four electrodes in the frequency ranging from 0.01 Hz to 10 kHz. (**e**) The enlarged part of (**d**). (**f**) Equivalent circuit model.

To evaluate the rate capability of MFe_2_O_4_/GNRs, the electrodes were cycled under various current densities from 0.2 to 10 A g^−1^, and the results were described in Figure 5a and Table S1. With the increase in current density, the specific capacity of the composites decreases. Because of the slow transportation of Li^+^ ions and insufficient lithiation-delithiation process, a number of “dead” Li^+^ ions are generated, leading to a decrease in the capacity of the electrodes. However, when the current density exceeded 5 A g^−1^, the capacity increased instead. This phenomenon was interpreted by the activation process to facilitate Li^+^ ion pathways between the electrolyte and the electrode under small current density cycling [42]. Meanwhile, the capacity of CoFe_2_O_4_, NiFe_2_O_4_, CoFe_2_O_4_/GNRs, and NiFe_2_O_4_/GNRs composites can be measured to 937, 867, 1475, and 1235 mA h g^−1^, respectively, when the current density returned to 0.2 A g^−1^, indicating high reversible rate capability. Even at the higher current density of 5 A g^−1^, the discharge capacities of CoFe_2_O_4_/GNRs and NiFe_2_O_4_/GNRs composite remain high at stable values of 1330 and 960 mAh g^−1^, respectively. However, pure CoFe_2_O_4_ and NiFe_2_O_4_ cannot maintain capacity in such a high rate due to the lack of GNRs skeleton. In addition, we discovered that the CoFe_2_O_4_/GNRs electrode shows better rate performance than NiFe_2_O_4_/GNRs, CoFe_2_O_4_, and NiFe_2_O_4_ electrode. The reason might be associated with more stable states and more negative adsorption energy of CoFe_2_O_4_/GNRs, which could produce extra capacity through the pseudo-capacitive behavior. Moreover, the larger BET surface area and more metal oxides component of CoFe_2_O_4_/GNRs contribute to better performance in the rate test and the following tests.

The cycling performance of CoFe_2_O_4_, NiFe_2_O_4_, CoFe_2_O_4_/GNRs, and NiFe_2_O_4_/GNRs electrodes at the current density of 0.1 A g^−1^ is presented in Figure 5b. The CoFe_2_O_4_/GNRs electrode expresses the best cycle performance after 80 discharge/charge cycles with a reversible capacity of 1432 mAh g^−1^. CoFe_2_O_4_ (715 mAh g^−1^), NiFe_2_O_4_ (569 mAh g^−1^), and NiFe_2_O_4_/GNRs (1085 mAh g^−1^) electrodes with relatively smaller specific capabilities exhibited cycle stability similar to CoFe_2_O_4_/GNRs. When we increased the current density to 1 A g^−1^, the results were shown in Figure 5c. After 100 cycles, the discharge capacity of CoFe_2_O_4_/GNRs and NiFe_2_O_4_/GNRs was 1720 mAh g^−1^ and 1414 mAh g^−1^, respectively, while CoFe_2_O_4_ and NiFe_2_O_4_ were 300 mAh g^−1^ and 201 mAh g^−1^, respectively. Compared to the previously reported MFe_2_O_4_ electrodes, the discharge capacity and cycle stability of the electrode materials were superior (Table S2). Furthermore, the capacities that increased gradually in the initial cycles could be the interfacial Li^+^ ion storage or the reversible polymer film produced by Co-, Ni-, and Fe-activated electrolyte degradation [32,42]. In the cycles, network GNRs can be used as the support of MFe_2_O_4_/GNRs, which can inhibit the volume changes in MFe_2_O_4_ during the charge-discharge process. In the meantime, the embedded MFe_2_O_4_ in the interlayers of GNRs would prevent the re-stacking and provide extra Li^+^ ion storage accommodation. Moreover, the transport channels in porous GNRs can speed up the conduction of electrons/ions. Figure S6 showed the SEM images of MFe_2_O_4_/GNRs electrodes after 100 cycles at 1A g^−1^, illustrating that the network remains intact in the process of cycles, which ensured excellent electrochemical performance. However, the CoFe_2_O_4_ and NiFe_2_O_4_ electrodes detached from the substrate and seriously agglomerated.

To obtain better insight into the mechanism of the electrode reactions in the unique MFe_2_O_4_/GNRs architectures, we performed electrochemical impedance spectroscopy (EIS) for both electrodes, as shown in Figure 5d,e. R_ct_, R_SEI_, and R_s_ correspond to the semicircle in the high-frequency region of typical Nyquist plots; CPE relates to the constant phase element [15] on the basis of equivalent electrical circuit mode. The R_ct_ of the CoFe_2_O_4_/GNRs and NiFe_2_O_4_/GNRs electrodes is 9.5 Ω and 16.8 Ω, respectively, which is smaller than CoFe_2_O_4_ and NiFe_2_O_4_ electrodes, showing that GNRs can effectively reduce the resistance of the anode. In the low-frequency region, the sloping line refers to the Warburg impedance (Zw); the slope of the CoFe_2_O_4_/GNRs electrode is higher than that of the NiFe_2_O_4_/GNRs, CoFe_2_O_4_, and NiFe_2_O_4_ electrodes, which implies that the CoFe_2_O_4_/GNRs electrode has lower Li^+^ ion diffusion resistance [31] (Table S3).

### 2.5. DFT Analysis

**Figure 6:**
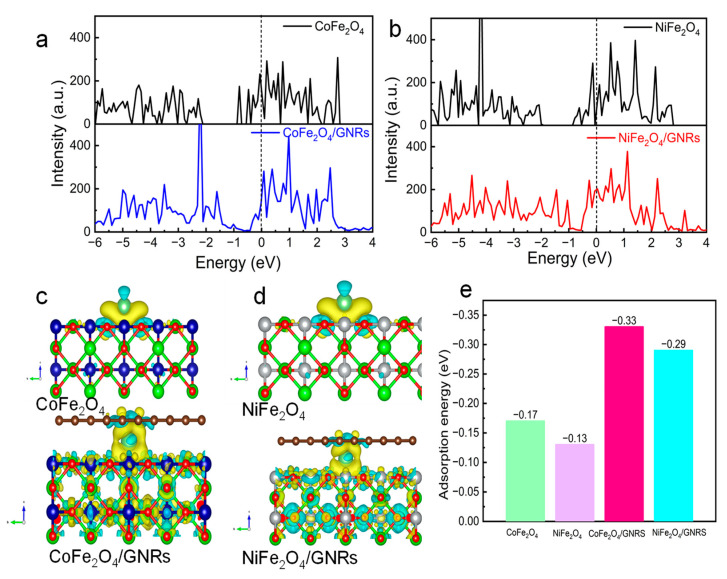
The calculated band gap of (**a**) CoFe_2_O_4_ and CoFe_2_O_4_/GNRs. (**b**) NiFe_2_O_4_ and NiFe_2_O_4_/GNRs. (**c**,**d**) Simulated adsorption configurations and (**e**) corresponding adsorption energy.

To further explain the effect of GNRs, we performed DFT calculations focused on the adsorption abilities towards Li^+^ ions and charge distribution after intercalation of Li^+^ ion [43,44]. CoFe_2_O_4_ and NiFe_2_O_4_ are semiconductors with band gaps of about 1.2 and 1.4 eV, respectively (Figure 6a–d). Since VASP is considered as a pseudopotential, the calculated value is relatively small. After the wrapping of GNRs, the hybrid orbitals appear near the Fermi level, leading to better electrical conductivity, which is consistent with the EIS results. According to Bader charge analysis, the number of transferred electrons for CoFe_2_O_4_ and NiFe_2_O_4_ is 0.23 and 0.26, respectively, while CoFe_2_O_4_/GNRs transfer 0.41 e^−^ and NiFe_2_O_4_/GNRs transfer 0.33 e^−^, indicating the more stable state of CoFe_2_O_4_/GNRs. Moreover, Li atoms adsorption models of MFe_2_O_4_ and Mfe_2_O_4_/GNRs were constructed to calculate the corresponding adsorption energy (ΔE_ads_). As can be seen in Figure 6e, the ΔE_ads_ of lithium adsorption in the CoFe_2_O_4_/GNRs is remarkably higher than that of NiFe_2_O_4_/GNRs, CoFe_2_O_4_, and NiFe_2_O_4_/GNRs, exhibiting that the CoFe_2_O_4_/GNRs can propel the lithium process. Accordingly, the theoretical analysis and experimental results are In a great consistence that the charge/discharge kinetics can be enhanced via GNRs assembling CoFe_2_O_4_ and NiFe_2_O_4_ to boost the electric conductivity and the advanced intrinsic Li^+^ storage.

The excellence electrochemical performance of MFe_2_O_4_/GNRs electrodes is probably due to the following reasons [17,45]: (1) as an elastic buffer layer, GNRs in the composites can not only avoid the volume expansion of CoFe_2_O_4_ and NiFe_2_O_4_ during lithiation and delithiation but also efficiently prevent the cracking or crumbling of anode materials [46,47]; (2) GNRs have premium electrical conductivity and are regarded as the electron transport path among CoFe_2_O_4_ and NiFe_2_O_4_, reducing the inner resistance of Li^+^ ion batteries; (3) the nano-sized active materials act as nanospacers to refrain the re-stacking of GNRs and therefore maintain their high active surface area, which is beneficial for enhancing the capacity of the composites.

### 2.6. Magnetic Performance Analysis

**Figure 7:**
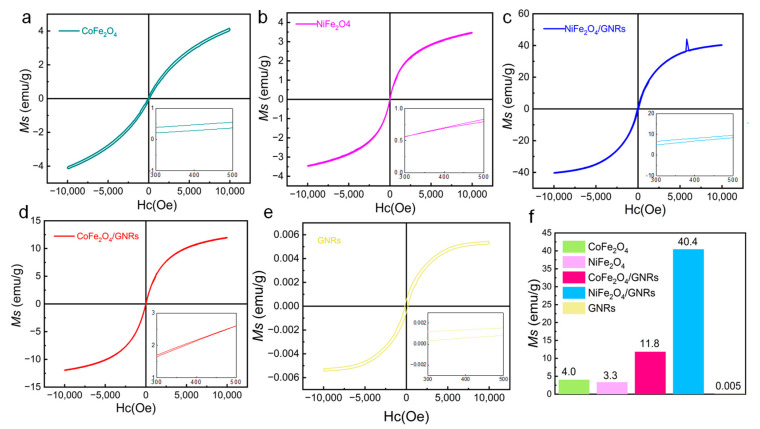
Hysteresis curves of (**a**) CoFe_2_O_4_, (**b**) NiFe_2_O_4_, (**c**) CoFe_2_O_4_/GNRs, (**d**) NiFe_2_O_4_/GNRs, and (**e**) GNRs (insets are the enlarged part of the samples); (**f**) saturation magnetization of as-prepared samples.

CoFe_2_O_4_ and NiFe_2_O_4_ nanoparticles are typical ferromagnetic nanomaterials. To clarify the effect of magnetism of CoFe_2_O_4_/GNRs and NiFe_2_O_4_/GNRs, the magnetic properties were studied by vibrating sample magnetometer system (VSM) at room temperature, as shown in Figure 7. Each CoFe_2_O_4_ and NiFe_2_O_4_ exhibited superparamagnetic behavior with little remanence and coercivity, implying that there is no remaining magnetization when the applied magnetic field is removed [48]. However, the saturation magnetization (Ms) values of CoFe_2_O_4_/GNRs and NiFe_2_O_4_/GNRs nanocomposites were 11.8 emu/g and 40.4 emu/g, respectively. The saturation magnetization intensity is lower than that of their bulk material. The main reason for the low saturation magnetization could be the size effect of magnetic nanoparticles; the surface spin coupling of nanoparticles is weaker than that of bulk materials. The saturation magnetization of all MFe_2_O_4_/GNRs composites is much higher than that of MFe_2_O_4_ and GNRs. It is noted that the saturation magnetization of NiFe_2_O_4_ nanoparticles in the NiFe_2_O_4_/GNRs is improved 10 times as much as that on their own. The improved saturation magnetization of NiFe_2_O_4_/GNRs is probably due to the increased electron conductivity by the aid of GNRs. In addition, we also calculated the magnetism enhancement after recombination of GNRs; the reason may be the influence of the built-in electromagnetic field on lithium storage (Table S4).

The CoFe_2_O_4_/GNRs and NiFe_2_O_4_/GNRs have maintained the dispersion and paramagnetism of magnetic nanoparticles, demonstrating their promising applications in magnetic devices.

## 3. Materials and Methods

### 3.1. Synthesis

The synthesis processes of GNRs and MFe_2_O_4_/GNRs were shown in supporting information [30].

### 3.2. Electrochemical Measurement

Coin cells (CR2430) were used to characterize the electrochemical properties of the sample. The working electrode was prepared by dropping the suspension of composites into a carbon nanofiber paper (CFP, d~16 mm, S~2 cm^2^) without any binder, then dried in vacuum. Cyclic voltammetry (CV) was carried out on an electrochemical workstation (Solartron Metrology 1260 + 1287, Bognor Regis, UK) with the scanning rate of 0.1 mV s^−1^ (0.00–3.00 V vs. Li/Li^+^). Arbin BT (2000, College Station, TX, USA) was used to measure charge–discharge properties of the samples. All the capacities showed in this work have deducted the contribution from the substrate.

### 3.3. Sample Characterization

X-ray diffraction (XRD, DMAX-2500PC, Tokyo, Japan) was used to record the phase structure of the samples using Cu/Kα radiation (kα = 0.15406 nm). Raman spectra were obtained by a Renishaw Invia Raman Microprobe (Wotton-under-Edge, UK) (100–3000 cm^−1^) using argon ion laser (514 nm). The morphologies of the as-obtained samples were studied using a field emission scanning electronic microscope (FE-SEM, Hitachi S-4800, Tokyo, Japan) and transmission electron microscope (TEM, Zeiss LIBRA 220 FEG, Oberkochen, Germany). X-ray photoelectron spectroscopy (XPS, ESCALAB 250 Thermo Fisher Scientific, Waltham, MA, USA) was carried out to study the surface chemical state of the composites. Brunner–Emmet–Teller (BET) measurements were operated on a Micromeritics ASAP 2020, USA. The content of the sample was analyzed by the thermogravimetric analysis (TGA Perkin ELMER TGA7, Waltham, MA, USA). The measurement of magnetic hysteresis loop was performed using a vibrating sample magnetometer (VSM Lake Shore 7400, Lake Shore Cryotronics, Westerville, OH, USA). All calculations were completed within the framework of the density functional theory (DFT) within the projector’s enhanced plane wave method.

## 4. Conclusions

A general magnetically induced self-assembled graphene nanoribbons method is proposed to prepare network MFe_2_O_4_/GNRs. The formation of these MFe_2_O_4_/GNRs is due to the surface in situ growth of MFe_2_O_4_ on GNRs and aggregation at the joints of GNRs, which is mainly driven by their own magnetic interactions. The unique framework of MFe_2_O_4_/GNRs can effectively prevent the shedding of MFe_2_O_4_, inhibiting the re-stacking of GNRs layers and shortening the Li+ ion transmission path. Consequently, CoFe_2_O_4_/GNRs and NiFe_2_O_4_/GNRs composites exhibited large reversible capacities (1432 and 1058 mAh g^−1^ at 0.1 A g^−1^, 1720 mAh g^−1^ and 1414 mAh g^−1^ at 1 A g^−1^, respectively), excellent cyclic performance, and good rate capabilities. The reason that the CoFe_2_O_4_/GNRs electrode shows better Li-ion battery performance than NiFe_2_O_4_/GNRs might be associated with the more stable states and more negative adsorption energy of CoFe_2_O_4_/GNRs according to DFT results, which could produce extra capacity through pseudo-capacitive behavior. Additionally, combining GNRs with MFe_2_O_4_ helps to improve the magnetism of the MFe_2_O_4_, making MFe_2_O_4_/GNRs suitable for magnetic applications, such as magnetically mediated targeted drug delivery and magnetic resonance imaging.

## Data Availability

The data that support the findings of this study are available from the authors.

## Supplementary Materials

The following supporting information can be downloaded at: https://www.mdpi.com/article/10.3390/molecules28104069/s1, 1 Synthesis of MFe_2_O_4_/GNRs. 2 Computational Method. Figure S1: (a,b) SEM images of as-prepared CoFe_2_O_4_; (b–d) SEM images of as-prepared NiFe_2_O_4_; Figure S2 (a–e) TEM images of as-prepared CoFe_2_O_4_/GNRs; Figure S3: The nitrogen adsorption/desorption isotherms and porosity distribution of (a,b) CoFe_2_O_4_/GNRs and (c,d) NiFe_2_O_4_/GNRs; Figure S4: TGA curves of CoFe_2_O_4_/GNRs and NiFe_2_O_4_/GNRs.; Figure S5 (a) CoFe_2_O_4_/GNRs and (b) NiFe_2_O_4_/GNRs electrodes at a current density of 0.1 A g^−1^ for the 1st, 2nd, 3rd, 30th, and 50th cycles in the voltage range between 3.00 and 0.01 V; Figure S6 SEM images of (a,b) CoFe_2_O_4_/GNRs and (c,d) NiFe_2_O_4_/GNRs electrode; Figure S7 SEM images of (a) CoFe_2_O_4_/GNRs, (b) NiFe_2_O_4_/GNRs, (c) CoFe_2_O_4_/GNRs, (d,e) CoFe_2_O_4_, and (f) NiFe_2_O_4_ electrode after 100 cycles (1 Ag^−1^). Table S1: Rate capability of the samples; Table S2: Comparison of the electrochemical performance of some Li-ion battery anodes; Table S3: The EIS simulation parameters of as-prepared samples; Table S4: Saturation magnetization of as-prepared samples. References [49,50,51] are cited in the supplementary materials.

## Sample Availability

Samples of the compounds are available from the authors.
