# Supplementary material for: Magnetic CoFe2O4 and NiFe2O4 Induced Self-Assembled Graphene Nanoribbon Framework with Excellent Properties for Li-Ion Battery
Source: Molecules. 2023 May 12;28(10):4069. doi: 10.3390/molecules28104069 (PMC10221835; doi:10.3390/molecules28104069)
Supplement: Supplementary file 1 [file molecules-28-04069-s001.zip › molecules-2369528-supplementary.pdf]

Supplementary Materials

Magnetic $CoFe_2O_4$ and $NiFe_2O_4$ Induced Self-Assembled Graphene Nanoribbon Framework with Excellent Properties for Li-Ion Battery

Xiyu Zhao [1,2], Chunyang He [1], Qiujv Bai [1], Xiangwen Miao [1], Cheng Cao [1] and Tianli Wu [2,*]

[1] School of Chemistry and Chemical Engineering, Key Laboratory of Hexi Corridor Resources Utilization of Gansu, Hexi University, Zhangye 734000, China
[2] Henan Key Laboratory of Photovoltaic Materials, Henan University, Kaifeng 475001, China
* Correspondence: tianliwu@cqu.edu.cn

1. Synthesis of MFe_2O_4/GNRs

GNRs were prepared by splitting multiwalled carbon nanotubes with Na/K in dimethoxyethane (DME), as described by James M. Tour. Iron (III) acetylacetonate (0.30g), cobalt (II) acetylacetonate (0.15 g), 1,2- dodecanediol (1.02 g), oleylamine (1.25 mL), oleic acid (0.9 mL), and GNRs (20 mg) were added in 20 mL benzyl ether under continuous stirring (200 rpm). Then, the mixture was refluxed at 220 °C for 30 min. After cooling to room temperature, the precipitates were collected by adding 20 mL methanol and centrifuging at 4000 rpm for 5 min. After centrifuging three times, the precipitates were dispersed in hexane (20 mL) solution. The $NiFe_2O_4$/GNRs is obtained by replacing the cobalt (II) acetylacetonate with the nickel (II) acetylacetonate at the same mass ratio. The $CoFe_2O_4$ and $NiFe_2O_4$ are obtained without GNRs.

2. Computational Method

The generalized gradient approximation proposed by Perdew, Burke, and Ernzerhof is chosen as the exchange correlation potential. The cut-off energy for plane wave is set to 500 eV. The energy criterion is set to 10−5 eV in iterative solution of the Kohn–Sham equation. A vacuum layer of 15 Å is added perpendicular to the sheet to avoid artificial interaction between periodic images. The Brillouin zone integration is performed using a 3x3x1 k-mesh. All the structures are relaxed until the residual forces on the atoms have declined to less than 0.01 eV/Å. All the calculations are performed in the framework of the density functional theory with the projector-augmented plane-wave method, as implemented in the Vienna ab initio simulation package. The generalized

gradient approximation proposed by Perdew, Burke, and Ernzerhof is selected for the exchange correlation potential. The cut-off energy for plane wave is set to 500 eV. The energy criterion is set to 10−5 eV in iterative solution of the Kohn–Sham equation. A vacuum layer of 15 Å is added perpendicular to the sheet to avoid artificial interaction between periodic images. The Brillouin zone integration is performed using a 3x3x1 k-mesh. All the structures are relaxed until the residual forces on the atoms have declined to less than 0.01 eV/Å.

DFT calculation details. The first-principles calculations were carried out with the Vienna ab initio simulation package1,2. The interaction between ions and valence electrons is described using projector-augmented wave (PAW) potentials, and the exchange correlation between electrons is treated through using the generalized gradient approximation (GGA) in the Perdew–Burke–Ernzerhof (PBE) form3. To achieve the accurate density of the electronic states, the plane wave cutoff energy was 500 eV, and a 3× 3×1 sheet k-point mesh was used. Ionic relaxations were carried out under the conventional energy (10-4 eV) and force (0.01 eV/Å) convergence criteria. The slab along the (001) projection was used to mimic the as-prepared (001) lattice plane, in which 15 Å vacuum layer was added to avoid the interaction between adjacent layers.

Gibbs free energies for each gaseous and adsorbed species were calculated at 298.15 K, according to the expression:

$$G = E_{DFT} + E_{ZPE} - TS$$

$$E_{ZPE} = \sum_i 1/2\ h\nu_i$$

$$\Theta_i = h\nu_i / k$$

$$S = \sum_i R[\ln (1-e^{-\Theta i/T})^{-1} + \Theta_i/T\ (e^{\Theta i/T} - 1)^{-1}]$$

where E_{DFT} is the electronic energy calculated for specified geometrical structures, E_{ZPE} is the zero-point energy, S is the entropy, h is the Planck constant, ν is the computed vibrational frequencies, Θ is the characteristic temperature of vibration, k is the Boltzmann constant, and R is the molar gas constant. For adsorbates, all 3N degrees of freedom were treated as frustrated harmonic vibrations with negligible contributions from the catalysts' surfaces. In the computational hydrogen electrode (CHE) model,[3] each reaction step was treated as a simultaneous transfer of the proton–electron pair as a function of the applied potential.

The adsorption energy (E_{ads}) of the OCHO was calculated as $E_{ads} = E_{substrate+adsorbate} - E_{substrate} - E_{adsorbate}$.

Figure S1 (a-b) SEM images of as-prepared $CoFe_2O_4$; (b-d) SEM images of as-prepared $NiFe_2O_4$.

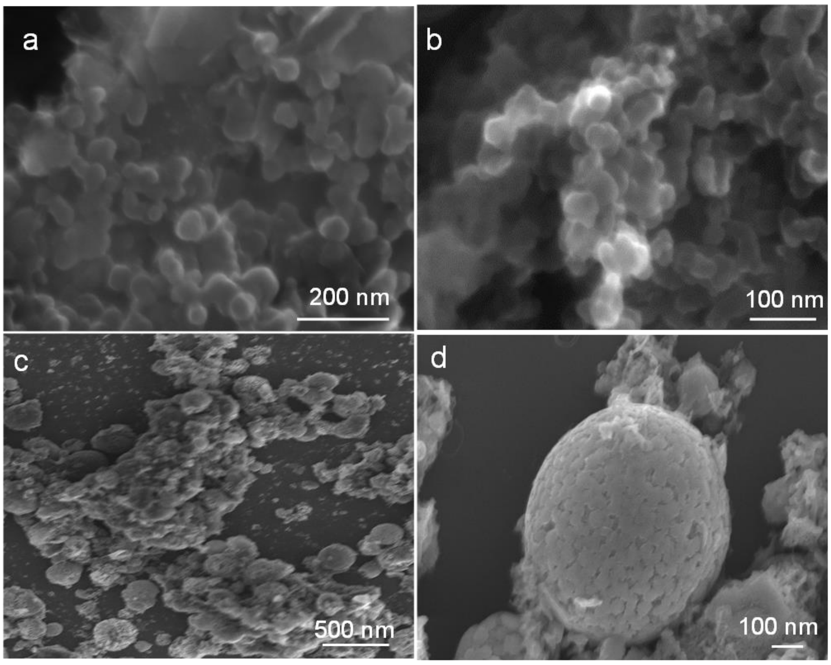

Figure S2 (a-e) TEM images of as-prepared $CoFe_2O_4$/GNRs.

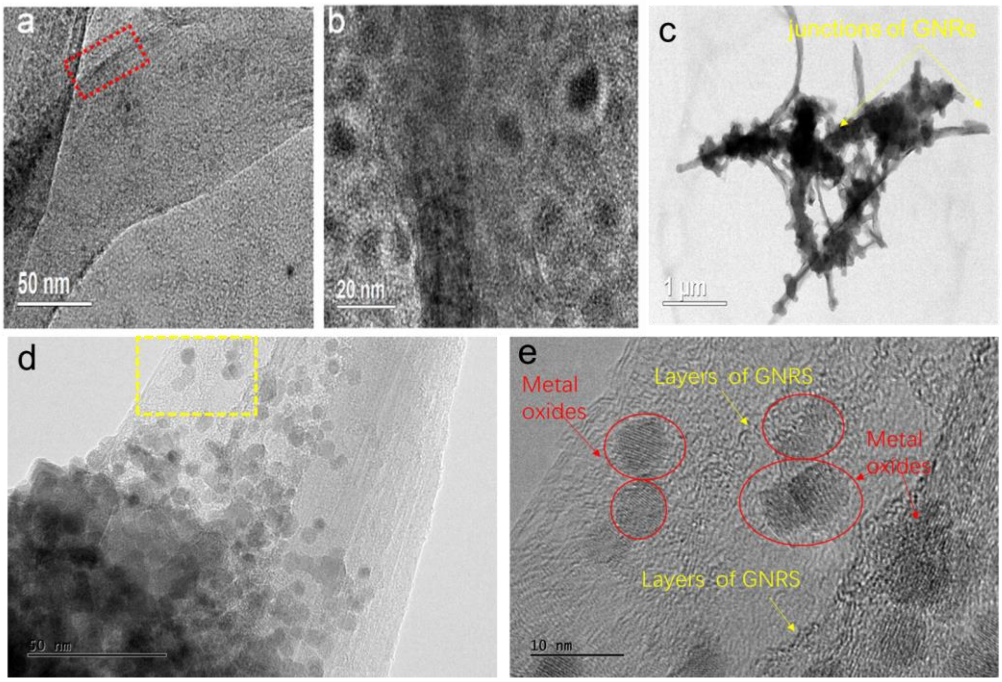

Figure S3 The nitrogen adsorption/desorption isotherms and porosity distribution of (a-b) $CoFe_2O_4$/GNRs and (c-d) $NiFe_2O_4$/GNRs.

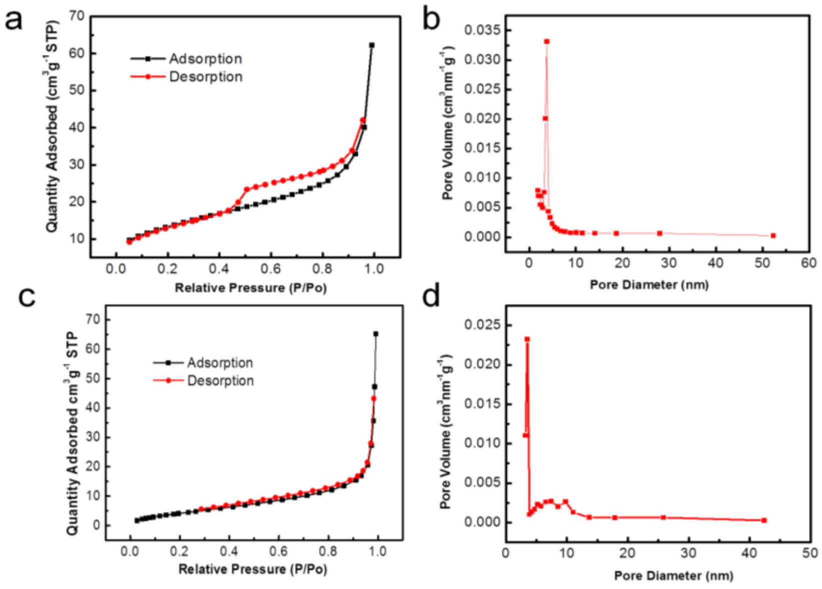

Figure S4 TGA curves of $CoFe_2O_4$/GNRs and $NiFe_2O_4$/GNRs.

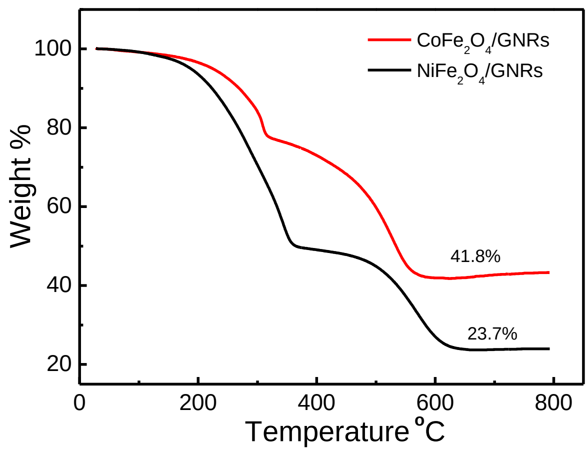

Figure S5 (a) $CoFe_2O_4$/GNRs and (b) $NiFe_2O_4$/GNRs electrodes at a current density of 0.1 A g^{-1} for the 1st, 2nd, 3rd, 30th, and 50th cycles in the voltage range between 3.00 and 0.01 V.

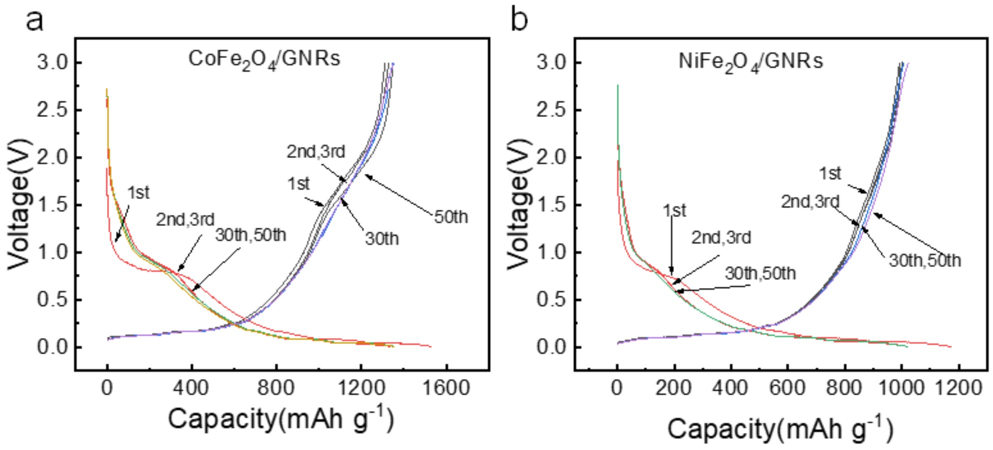

Figure S6 SEM images of (a-b) $CoFe_2O_4$/GNRs and (c-d) $NiFe_2O_4$/GNRs electrode.

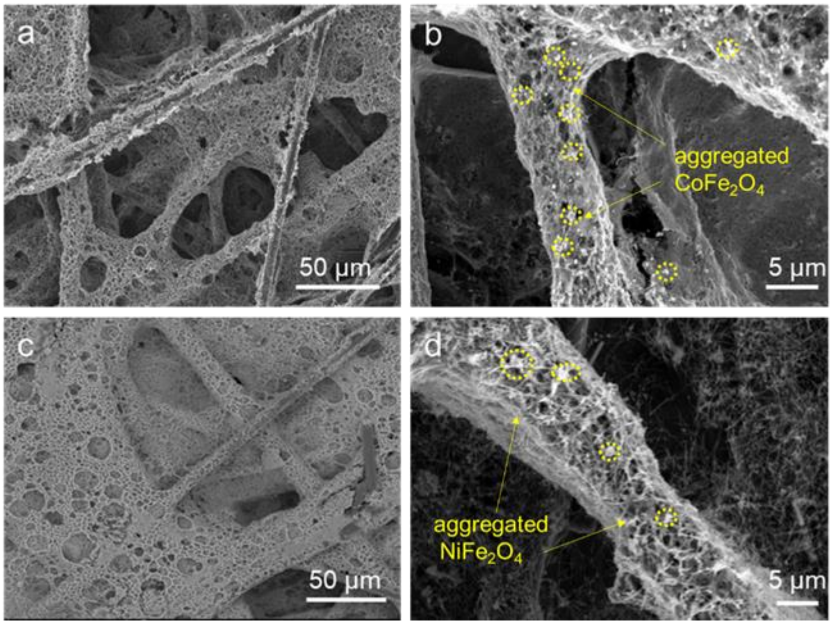

Figure S7 SEM images of (a) $CoFe_2O_4$/GNRs, (b) $NiFe_2O_4$/GNRs, (c) $CoFe_2O_4$/GNRs, (d-e) $CoFe_2O_4$, and (f) $NiFe_2O_4$ electrode after 100 cycles (1 Ag^{-1}).

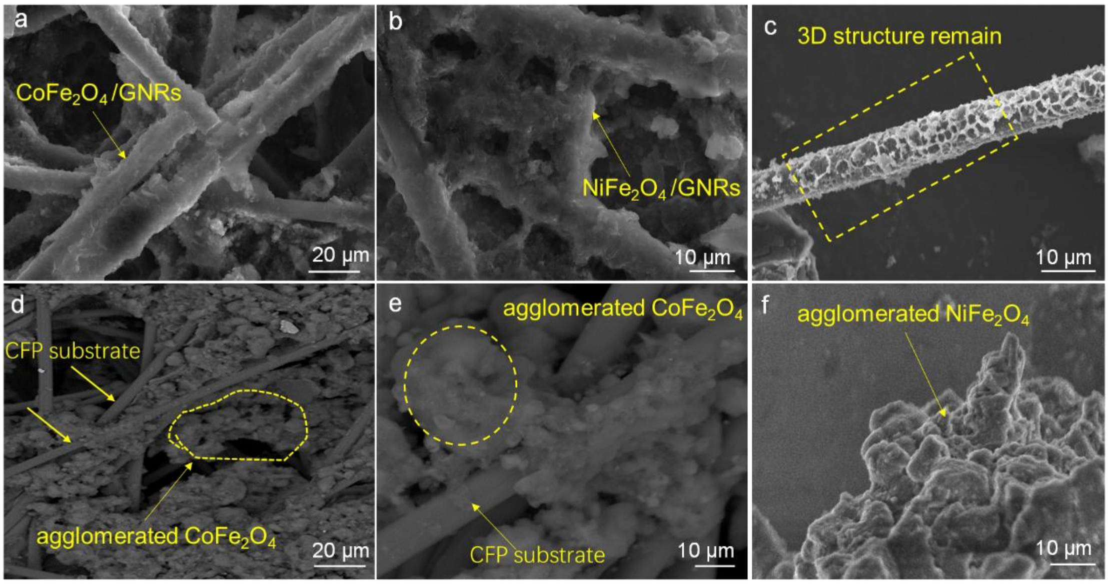

Table S1 Rate capability of the samples.

Current density (A g^{-1}) / Capacity (mAh g^{-1})	0.2	0.5	1.0	2.0	5.0	10.0	0.2
$CoFe_2O_4$/	873	900	694	238	187	0	937
$NiFe_2O_4$/	758	847	603	193	116	0	867
$CoFe_2O_4$/GNRs	1378	1083	1119	1295	1330	468	1475
$NiFe_2O_4$/GNRs	1163	1031	923	948	960	273	1235

Table S2 Comparison of the electrochemical performance of some Li-ion battery anodes.

Samples	Current density (A g^{-1})	Cycle number (Cycles)	Capacity (mAh g^{-1})	References
$CoFe_2O_4$/C	0.1	50	227	*ChemistrySelect*,**2020**, *5*, 8207.
$CoFe_2O_4$/OLC	0.1	500	914.2	*J. Alloys Compd.*, **2015**, *644*, 59.
C@$CoFe_2O_4$ fiber-in-tube	0.2	200	740	*J. Alloys Compd.*, **2017**, *693*, 110.
$CoFe_2O_4$@CNT	0.1	60	243	*J. Alloys Compd.*, **2020**, 834, 155018.
$CoFe_2O_4$/CN-NG	0.1	50	918	*Nano Energy*, **2019**, *57*, 48.
$MnFe_2O_4$@C	1	400	850	*Nanoscale*, **2020**, *12* 4445.
H-$MnFe_2O_4$-C	0.2	500	973	*Chem. Eng. J.*, **2019**, *365*, 121.
$NiFe_2O_4$/C	0.1	50	892.4	*Electron. Mater. Lett.*, **2020**, *16*, 207.
$NiFe_2O_4$-rGO	0.2	300	1129	*Inorg. Chem. Front.*, **2019**, *6*, 961.
$ZnFe_2O_4$@C NTs	0.5	500	817.6	*Nanoscale Adv.*,**2020**, *2*, 2284.
**$CoFe_2O_4$/GNRs**	**0.1**<br>**1**	**80**<br>**100**	**1432**<br>**1720**	**This work**
**$NiFe_2O_4$/GNRs**	**0.1**<br>**1**	**80**<br>**100**	**1058**<br>**1414**	**This work**

Table S3 The EIS simulation parameters of as-prepared samples.

Sample	Rs(Ω)	R_{SEI}(Ω)	Rct(Ω)
$CoFe_2O_4$	9.5	10.7	20.3
$NiFe_2O_4$	7.5	27.5	35.9
$CoFe_2O_4$/GNRs	3.0	5.1	9.5
$NiFe_2O_4$/GNRs	3.5	9.5	16.8

Table S4 Saturation magnetization of as-prepared samples.

samples	$CoFe_2O_4$	$NiFe_2O_4$	$CoFe_2O_4$/GNRs	$NiFe_2O_4$/GNRs
Ms/emu g^{-1}	4.0	3.3	11.8	40.4
Calculated magnetism	126.57	101.93	157.69	110.01

References

1. Kresse, G.; Furthmuller, J. Efficiency of ab-initio total energy calculations for metals and semiconductors using a plane-wave basis set. *Comp. Mater. Sci.* **1996**, *6*, 15–50.

50. Surendranath, Y.; Kanan, M.W.; Nocera, D.G. Mechanistic studies of the oxygen evolution reaction by a cobalt-phosphate cata lyst at neutral pH. *J. Am. Chem. Soc*. **2010**, *132*, 16501–16509.

51. Peterson, A.A.; Abild-Pedersen, F.; Studt, F.; Rossmeisl, J.; Nørskov, J.K. How copper catalyzes the electroreduction of carbon dioxide into hydrocarbon fuels. *Energy Environ. Sci.* **2010**, *3*, 1311.
